# The Redox State of Transglutaminase 2 Controls Arterial Remodeling

**DOI:** 10.1371/journal.pone.0023067

**Published:** 2011-08-25

**Authors:** Jeroen van den Akker, Ed VanBavel, Remon van Geel, Hanke L. Matlung, Bilge Guvenc Tuna, George M. C. Janssen, Peter A. van Veelen, Wilbert C. Boelens, Jo G. R. De Mey, Erik N. T. P. Bakker

**Affiliations:** 1 Department of Biomedical Engineering and Physics, Academic Medical Center, University of Amsterdam, Amsterdam, The Netherlands; 2 Department of Biomolecular Chemistry 271, Nijmegen Center for Molecular Life Sciences, Radboud University, Nijmegen, The Netherlands; 3 Department of Immunohematology and Blood Transfusion, Leiden University Medical Centre, Leiden, The Netherlands; 4 Netherlands Proteomics Centre, Utrecht, The Netherlands; 5 Department of Pharmacology and Toxicology, Cardiovascular Research Institute Maastricht, Maastricht University, Maastricht, The Netherlands; King's College London, University of London, United Kingdom

## Abstract

While inward remodeling of small arteries in response to low blood flow, hypertension, and chronic vasoconstriction depends on type 2 transglutaminase (TG2), the mechanisms of action have remained unresolved. We studied the regulation of TG2 activity, its (sub) cellular localization, substrates, and its specific mode of action during small artery inward remodeling. We found that inward remodeling of isolated mouse mesenteric arteries by exogenous TG2 required the presence of a reducing agent. The effect of TG2 depended on its cross-linking activity, as indicated by the lack of effect of mutant TG2. The cell-permeable reducing agent DTT, but not the cell-impermeable reducing agent TCEP, induced translocation of endogenous TG2 and high membrane-bound transglutaminase activity. This coincided with inward remodeling, characterized by a stiffening of the artery. The remodeling could be inhibited by a TG2 inhibitor and by the nitric oxide donor, SNAP. Using a pull-down assay and mass spectrometry, 21 proteins were identified as TG2 cross-linking substrates, including fibronectin, collagen and nidogen. Inward remodeling induced by low blood flow was associated with the upregulation of several anti-oxidant proteins, notably glutathione-S-transferase, and selenoprotein P. In conclusion, these results show that a reduced state induces smooth muscle membrane-bound TG2 activity. Inward remodeling results from the cross-linking of vicinal matrix proteins, causing a stiffening of the arterial wall.

## Introduction

Small arteries represent the main site of resistance in the vascular system, and as such, have a large impact on tissue perfusion and blood pressure. Inward remodeling of small arteries occurs after a reduction in blood flow, but is also associated with high blood pressure [1]–[3]. It is a hallmark of essential hypertension and a strong predictor of cardiovascular events [4]. Previous work from our group showed that transglutaminases, in particular type 2 transglutaminase (TG2), play a crucial role in the inward remodeling of small arteries after reduced blood flow and hypertension *in vivo*, and chronic vasoconstriction *in vitro* [5]–[11]. The mechanism by which TG2 contributes to vascular remodeling however, remains poorly understood.

One reason for the elusive role of TG2 in remodeling may relate to its wide range of actions. Its best known function is the stabilization of matrix proteins through the formation of a specific cross-link, the N^ε^(γ-glutamyl)lysine isopeptide bond, which is the result of a transamidation reaction [12]. This reaction is regulated by several factors including calcium, GTP, nitric oxide and the redox potential [13], [14]. The isopeptide bond provides mechanical strength and resistance to proteolytic degradation in tissues. Recent work from Santhanam et al [15] showed that the cross-linking action of TG2 is highly relevant for human cardiovascular pathology. Thus, these authors showed that the formation of cross-links by TG2 relates to the stiffening of large arteries that is associated with aging. Besides the formation of isopeptide bonds within and between proteins [12], TG2 promotes cell adhesion via its binding to fibronectin, integrin α_5_β_1_ and heparan sulfate proteoglycans [16], [17]. In addition, TG2 acts as protein disulfide isomerase [18], functions as a G-protein [19] and aids in the regulation of cytoskeletal structure and cell contractility [20]–[22].

Of great potential relevance for cardiovascular pathology, Stamnaes et al. [23] recently showed that TG2 contains a triad of cysteine residues that act as a redox sensor, which could regulate the cross-linking activity of TG2 in the extracellular environment. In the present study we investigated the activation, (sub) cellular localization, and substrates of TG2 during the inward remodeling of small arteries. Besides the regulation of TG2 by calcium and nitric oxide, we focused on the redox state of TG2 using reducing agents with different properties regarding cellular permeability. We report that TG2 is translocated to the surface of smooth muscle cells (SMCs) upon intracellular reduction. In a reduced state TG2 acts as a membrane-bound cross-linking enzyme in SMC, which coincides with inward remodeling. Using mass spectrometry, we identified several matrix proteins as TG2 substrates. Taken together, these data elucidate the regulation, secretion and substrates of TG2 in the process of vascular remodeling.

## Methods

### Mice and vessel isolation

Four months old male C57Bl/6 mice (Harlan) were anesthetized using isoflurane and sacrificed by cervical dislocation. Then, the abdomen was opened and the mesentery was excised and placed in cold MOPS buffer. First and second order arteries were isolated from the mesenteric vasculature. Arteries were cut in equal-sized pieces where one segment was randomly assigned as control and the other segments subjected to various interventions. This approach allowed for pair-wise statistics and drastically decreased variability which results from anatomical variation in vessel caliber. All protocols consisted of a 24-hour incubation period at 37°C, where the vessels were placed in 100 µL buffer containing Leibovitz medium with 10% fetal bovine serum (Gibco), a mix of antibiotic-antimycotic solution (Gibco), and additional compounds, depending on the specific protocol. All experiments were approved by the Committee for Animal Experiments of the Academic Medical Center Amsterdam (permissions 101221 and 101555). TG2 knockout mice were obtained from Prof. G. Melino (Rome, Italy) and bred at our local facility.

### Pressure myograph and remodeling

To determine remodeling of the arteries, segments were cannulated in a pressure myograph system after the incubation period and inner diameters were recorded as described previously [11]. After checking for leaks, a passive pressure- diameter relationship was determined in calcium-free MOPS buffer, supplemented with papaverine (0.1 mmol/L) to rule out influences of vasomotor tone.

### Exogenous recombinant TG2

In this set of experiments, vessels were exposed to recombinant human TG2 (Zedira, T002) or cross-linking deficient C277S-TG2 (Zedira, T018), with or without the membrane-impermeable reducing agent tris(2-carboxyethyl)phosphine hydrochloride (TCEP, 1 mmol/L). Control experiments were included were segments only were exposed to TCEP. In all experiments, recombinant TG2 was administered at 50 µg/mL.

### Activation of endogenous TG2

In this set of experiments, vessels were incubated with calcium ionophore A23187 (Sigma, C7522: 1 µmol/L). Alternatively, vessels received 2 mmol/L DTT (Sigma, 43816), which is membrane-permeable. In the latter experiment, TG2 activity was blocked using either the TG2 active-site inhibitor L682777 (Zedira, T101: 10 µmol/L, also known as R283) or the NO donor SNAP (Sigma, N3398: 1 mmol/L).

The effect of DTT on vessel viability was assessed in a separate set of vessels. Here, the contractile response to the thromboxane/prostaglandin agonist U46619 (Sigma, D8174: 1 µmol/L) was measured after a 24-hour incubation with 2 mmol/L DTT.

### Localization of TG2 activity

In vessels stimulated with calcium ionophore or DTT, TG2 activity was visualized using the pseudo-substrate cadaverine, linked to either FITC (AnaSpec, 81504; 100 µmol/L) or AlexaFluor594 (Invitrogen, A-30678;10 µmol/L). In experiments where SNAP was used to inhibit TG2 activity, cadaverine was added >30 min after SNAP. SNAP was used at 10^−4^ mol/L with calcium ionophore and at a concentration of 10^−3^ mol/L with DTT. Vessels were fixed with formalin, mounted on glass slides using Vectashield/DAPI (Vector Laboratories H-1500) and imaged on a confocal microscope (Leica TCS SP2). TG2 activity was quantified by spatial integration of FITC or AlexaFluor594 signal in ImageJ. Data were corrected for vessel size and depicted in arbitrary units.

### Immunostaining of TG2

The effect of DTT on the translocation of TG2 was assessed by immunofluorescent staining of extracellular TG2 on cultured mouse smooth muscle cells (MOVAS, ATCC CRL-2797). Cells were grown in microscopic culture chambers (BD Falcon 354102, untreated glass) that were coated with fibronectin. After a culture period of 24 hours in DMEM with 10% FCS, 0.1 mmol/L DTT was added for 2 hrs. Then cells were washed 3 times with warm PBS and fixated with cold formalin. After blocking with BSA/goat serum, the non-permeabilized cells were stained with a rabbit polyclonal TG2 antibody Ab-4 (Neomarkers RB-060-P, 1∶10; 1 hr at room temperature) followed by anti-rabbit Cy3 (Brunschwig 111-165-144, 1∶200; 1 hr at room temperature) as secondary antibody, and slides were mounted in Vectashield/DAPI (Vector Laboratories H-1500).

### Substrates of transglutaminase in smooth muscle cells

The substrates for transamidation catalyzed by TG2 were determined using a mouse smooth muscle cell line (MOVAS, ATCC CRL-2797). Cells were cultured in DMEM with 10% FCS for 96 hours in T75 flasks. Then either BPA (biotinylated pentylamine, Invitrogen A1594, 1 mmol/L) or Q-peptide (Biotin-GQEPVR, synthesized using standard Fmoc-based solid phase peptide synthesis, 0.25 mmol/L) were added to function as lysine and glutamine donors respectively [24], while a control group was left without competitive substrate. All groups received 0.1 mmol/L DTT to increase the amount of active TG2 in the extracellular matrix. After a 24 hrs incubation period, the cultures were washed 3 times with warm PBS to remove non-bound BPA and Q-peptide. Then the lysates were collected in a 1% SDS solution and boiled for 5 minutes at 95°C to denature the proteins. For each group, the material from 2 T75 flasks was pooled and stored at −20°C until further use.

Lysates were sonicated (5 times 30 seconds at room temperature) and dialysed against 1% SDS (3 times 500 mL for 1 hour at room temperature) to further decrease the amount of non-bound BPA and Q-peptide. Then lysates were centrifuged at 15.700 g for 10 minutes at room temperature and 13 mL buffer containing 100 mmol/L NaCl, 50 mmol/L Tris HCl pH 7.5, 1 mmol/L EDTA, 0.5% NP40 was added to 1.2 mL supernatant. Streptavidin-Agarose (50 µL, Sigma S1638) was added and the mixture was rotated end-over-end at room temperature for 20 hours. The agarose-beads were washed with 100 mmol/L NaCl, 50 mmol/L Tris HCl pH 7.5, 1 mmol/L EDTA, 0.05% NP40 (3 times 5 minutes end-over-end at room temperature) and taken up in 2x sample buffer (4% SDS, 10% β-mercaptoethanol, 20% glycerol, 0.06% bromophenol blue and 0.5 mol/L Tris HCl pH 6.8).

For Western blot analysis 2% of each pull-down was loaded on a 12% SDS-PAGE gel and after blotting stained with IRdye 800CW Streptavidin (li-Cor), rabbit polyclonal anti-Fibronectin (GIBCO 1A0540) and rabbit polyclonal nidogen-1 (Immun Diagnostik AP1003.1).

For MS analysis 20% of each pull-down was loaded on a 12% SDS-PAGE gel and stained with colloidal Coomassie Brilliant Blue. The biotin containing region (∼30 kDa and up) from each Coomassie stained lane was sliced into 16 equal parts. Proteins in the slice were reduced with DTT, alkylated with iodoacetamide and digested with trypsin using the Proteineer DP digestion robot (Bruker, Bremen, Germany), adapted in house to accommodate larger gel pieces. The tryptic peptides were extracted from the gel, lyophilized, dissolved in 95/3/0.1 v/v/v water/acetonitril/formic acid and subsequently analyzed by on-line nanoHPLC MS/MS using a1100 HPLC system (Agilent Technologies), as previously described [25]. Peptides were trapped at 10 µL/min on a 15-mm column (100-µm ID; ReproSil-Pur C18-AQ, 3 µm, Dr. Maisch GmbH) and eluted to a 200 mm column (50-µm ID; ReproSil-Pur C18-AQ, 3 µm) at 150 nL/min. All columns were packed in house. The column was developed with a 120-min gradient from 0 to 30% acetonitrile in 0.1% formic acid. The end of the nanocolumn was drawn to a tip (ID ∼5 µm), from which the eluent was sprayed into a 7-tesla LTQ-FT Ultra mass spectrometer (Thermo Electron). The mass spectrometer was operated in data-dependent mode, automatically switching between MS and MS/MS acquisition. Full scan MS spectra were acquired in the FT-ICR with a resolution of 25,000 at a target value of 3,000,000. The two most intense ions were then isolated for accurate mass measurements by a selected ion monitoring scan in FT-ICR with a resolution of 50,000 at a target accumulation value of 50,000. Selected ions were fragmented in the linear ion trap using collision-induced dissociation at a target value of 10,000. In a post-analysis process, raw data were first converted to peak lists using Bioworks Browser software v 3.2 (Thermo Electron), then submitted to the SwissProt database version 51.6 using Mascot v. 2.2.04 (www.matrixscience.com) for protein identification and finally sorted and compared using Scaffold software version 3.0.1 (www.proteomesoftware.com). Mascot searches were with 2 ppm and 0.8 Da deviation for precursor and fragment mass, respectively, and trypsin as enzyme. Scaffold filtered for identified proteins with at least 2 peptides with 95% confidence. Collision-induced dissociation spectra were also manually inspected. Common contaminants were removed manually from the list.

### Regulators of redox balance during inward remodeling

The expression of redox regulating enzymes in inwardly remodeling vessels was investigated using a microarray approach published previously by Wesselman et al. [26] In short, flow-modifying surgery was performed on rat first-order mesenteric arteries. This leads to a flow reduction in ligated vessels to approximately 10% of control, and a doubling of flow in adjacent high flow vessels. Animals were sacrificed after 1, 2, or 4 days and for each time point vessels from 4 animals were pooled. cDNA from either low or high flow vessels linked to a Cy5 probe was hybridized onto 2 different microarrays in the presence of Cy3-labeled cDNA from control vessels. Up- or down regulation of a number of redox regulating enzymes with decreased flow was then normalized to control values. If genes were present more than once in the 2 arrays employed, their values were averaged.

### Statistics

Data are shown as mean ± SEM. For all measurements of P,d-curves, differences in diameter between groups were tested at each pressure level using a paired T-test with Bonferroni correction when appropriate. Distensibility was calculated as the lumen diameter of the artery at 120 mmHg divided by the diameter at 5 mmHg. For quantification of fluorescence, 3 images were averaged per vessel. In all figures, P-values smaller than 0.05 resp. 0.01 are indicated by single or double symbols (e.g. * and **).

## Results

### Vascular remodeling by recombinant TG2 requires a reducing agent

**Figure 1:**
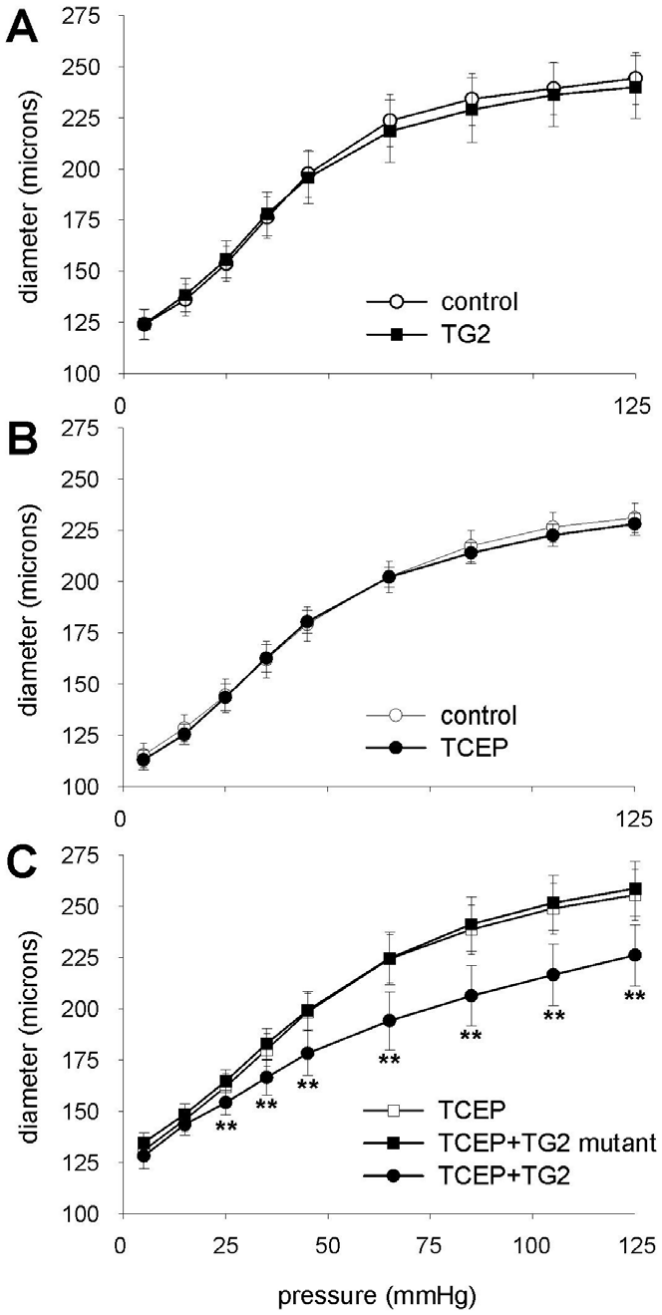
Microvascular remodeling by transglutaminase requires a reducing agent and cross-linking activity. Passive pressure-diameter relationships (P,d curve) of isolated, cannulated arteries were measured to reveal remodeling. (A) Exogenous TG2 had no effect on vessel properties in the absence of a reducing agent. (B) The cell-impermeable reducing agent TCEP did not induce remodeling by itself. (C) When exogenous TG2 was combined with TCEP, inward remodeling was observed. This effect was absent with recombinant TG2, defective in cross-linking. Data were averaged over 6 vessels obtained from 3 mice. ** TCEP vs. TG2+TCEP: P<0.01.

Small mesenteric arteries were incubated for 24 hours under various conditions. After the incubation period, vascular remodeling of these vessels was determined by cannulation and recording of a passive pressure-diameter (P,d) relationship. Incubation of mesenteric arteries with exogenous recombinant TG2 had no effect on the P,d-curve as compared to untreated control arteries (Figure 1A). Distensibility of the arteries, determined as the ratio of the diameter at 120 mmHg divided by the diameter at 5 mmHg, was unchanged: 1.98±0.14 vs. 1.93±0.10 for control and TG2 respectively. Administration of 1 mmol/L TCEP, a cell-impermeable reducing agent, also did not affect the P,d curve (Figure 1B). Distensibility was unchanged: 2.01±0.12 vs. 2.03±0.11 for control and TCEP respectively. However, when TG2 was added together with TCEP, vessel diameter was significantly reduced at higher pressure levels (Figure 1C). This resulted in a significant decrease in distensibility from 1.95±0.04 to 1.76±0.14 for TCEP vs. TCEP+TG2 (p = 0.01). When the catalytically inactive TG2 mutant Cys-277 was used instead of recombinant TG2, inward remodeling was again absent (Figure 1C). Distensibility was similar to TCEP alone: 1.92±0.08 (NS). These data therefore show that inward remodeling by TG2, characterized by a reduction in distensibility, depends on its cross-linking action and requires the presence of a reducing agent.

### A cell-permeable reducing agent activates endogenous TG2

**Figure 2:**
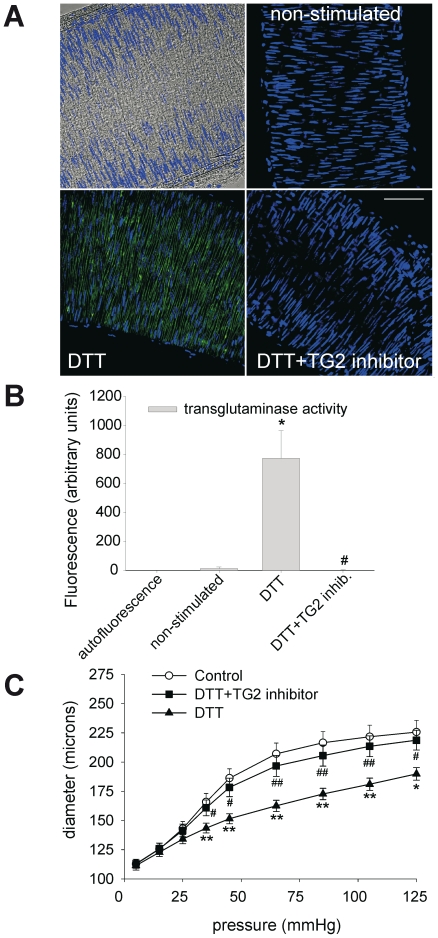
Activation of endogenous TG2 by the cell-permeable reducing agent DTT. (A–B) Exposure to DTT induces TG2 activity in the medial layer of the vessel, as shown by the incorporation of FITC cadaverine. (C) TG2 activation with DTT induces inward remodeling, as indicated by a downward shift of the P,d-curve. This was blocked with a site-specific TG2 inhibitor (L682777). Data were averaged over 6 vessels obtained from 3 mice, with 3 images per vessel; scalebar = 75 µm. * non-stimulated vs. DTT: P<0.05, ** P<0.01, # DTT vs. L682777+DTT: P<0.05, ## P<0.01.

In order to test if endogenous TG2 could be activated by a cell-permeable reducing agent, vessels were incubated with dithiothreitol (DTT). As indicated by FITC-cadaverine incorporation, DTT induced a profound increase in TG2 activity in the vessel wall (Figure 2A). This activity was completely abolished in vessels incubated with the TG2 inhibitor L682777 (Figure 2, panels A–B). The activation of endogenous TG2 with DTT caused a highly significant inward remodeling, which was almost completely prevented by L682777 (Figure 2C). The effect of endogenous TG2 on vascular remodeling was similar to that of exogenous TG2, with a reduction in vessel diameter at higher distending pressures only (Figure 2C). Thus, distensibility decreased from 2.00±0.12 to 1.71±0.08 for control and DTT respectively (p<0.01).

When small mesenteric arteries from TG2 knockout mice were exposed to DTT, inward remodeling was observed also, albeit to a lesser extent as compared to vessels from C57BL/6 mice. Distensibility decreased significantly from 2.10±0.08 to 1.95±0.08 for control and DTT respectively (p<0.001). In this case however, the TG2 inhibitor L682777 was ineffective. Distensibility was not altered as compared to DTT: 1.97±0.11 (NS). These data suggest that in the TG2 knockout mice other mechanisms are activated by DTT which induce remodeling (Figure S1).

**Figure 3:**
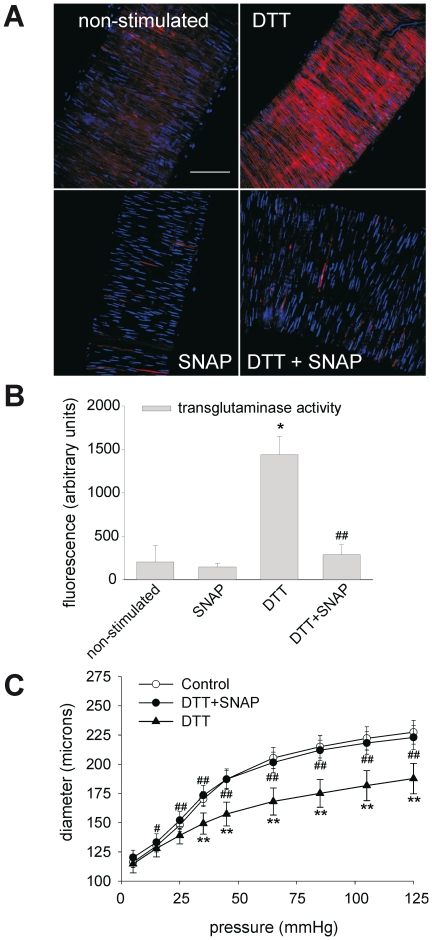
DTT-induced activation of TG2 can be counteracted with the NO donor SNAP. (A–B) Incorporation of AlexaFluor594/cadaverine was significantly inhibited with SNAP. (C) SNAP abolished the inward remodeling induced by DTT. Data were averaged over 6 vessels obtained from 3 mice, with 3 images per vessel. Scalebar = 75 µm. * non-stimulated vs. DTT: P<0.05, ** P<0.01, # DTT vs. SNAP+DTT: P<0.05, ## P<0.01.

In subsequent experiments we tested if remodeling induced by DTT could also be counteracted by nitric oxide. Transglutaminase activity was strongly reduced by the NO donor SNAP, as indicated by the incorporation of Alexa Fluor-594/cadaverine (Figure 3, panels A–B). This was paralleled by an almost complete inhibition of the remodeling (Figure 3C). Distensibility decreased significantly by treatment with DTT from 1.96±0.10 to 1.64±0.13 (p<0.01), but was reversed by SNAP to 1.86±0.06 (p = 0.01). Control experiments showed that SNAP alone did not affect vessel properties (Figure S2). Hence, these results show that a shift of the intracellular redox balance to a more reduced state activates a TG2-dependent inward remodeling and, in addition, this remodeling can be inhibited by nitric oxide (NO).

### Calcium ionophore A23187 stimulates TG2 activity but does not induce inward remodeling

**Figure 4:**
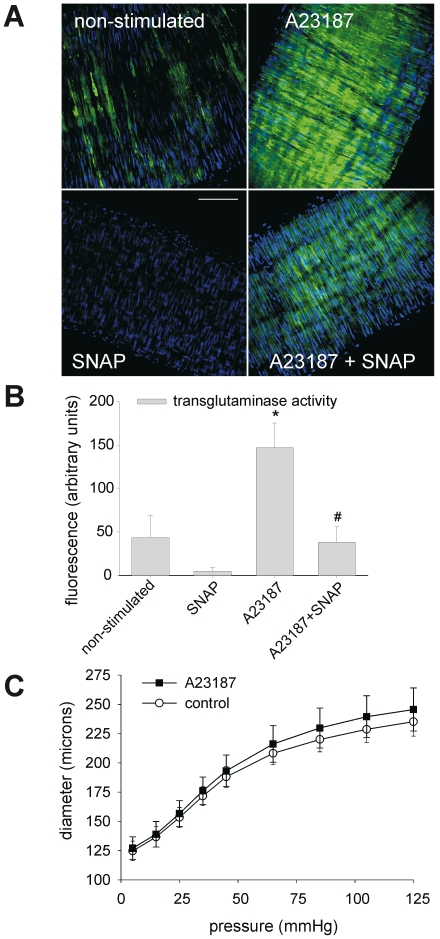
Incubation with calcium ionophore (A23187) induces transglutaminase activity without remodeling. (A–B) Exposure to A23187 stimulates the incorporation of FITC cadaverine. TG2 activity can be inhibited by the NO donor SNAP. (C) A23187 does not induce inward remodeling. Data were averaged over 6 vessels obtained from 3 mice, with 3 images per vessel. Scalebar = 75 µm. * non-stimulated vs. A23187: P<0.05, # A23187 vs. A23187+SNAP: P<0.05.

Calcium triggers a conformational change in TG2, providing access of substrates to TG2's active site [14]. Normally, under physiological conditions the level of intracellular calcium is too low to induce cross-linking activity by TG2. However, after exposure to vasoconstrictor substances, or under pathological conditions, the level of intracellular calcium may rise considerably. To test the role of calcium we analyzed the effect of the calcium ionophore A23187 on TG2 activity and arterial remodeling. Administration of the ionophore increased TG2 activity in the vessel wall about 3-fold, as demonstrated by the incorporation of FITC-cadaverine (Figure 4, panels A,B). This calcium-induced intracellular activity could be inhibited by 1 mmol/L of the NO donor SNAP (Fig. 4, panels A,B). Despite the increase in TG2 activity, the calcium ionophore did not induce small artery inward remodeling, as reflected by an unchanged P-d curve after incubation of cannulated arteries with 1 µmol/L A23187 (Figure 4C). Distensibility was also unchanged: 1.90±0.14 vs. 1.94±0.13 for control and A23187 respectively (NS).

### Localization of TG2 activity is stimulus-dependent

**Figure 5:**
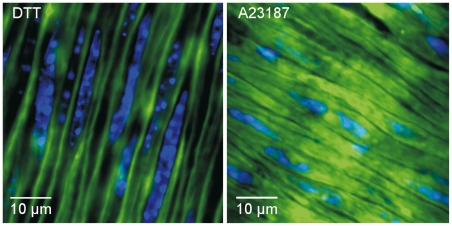
Differential localization of transglutaminase activity. (A) When vessels were incubated with the cell-permeable reducing agent DTT, FITC cadaverine was cross-linked at the cell membrane of smooth muscle cells. (B) When vessels were incubated with the calcium ionophore A23187, FITC cadaverine appeared throughout the cytosol. Vascular remodeling occurred with DTT treatment, but not with the calcium ionophore.

While activation of intracellular TG2 with DTT caused significant inward remodeling, this was absent for activation by the calcium ionophore A23187. We tested whether this difference is related to localization of TG2 activity. In the adventitia, TG2 activity was virtually absent (Video S1). The incorporation of FITC-cadaverine around endothelial cells was also relatively low. In contrast, TG2 activity following stimulation with A23187 or DTT was prominent in the medial layer. The subcellular staining pattern in the SMCs was strongly dependent on the stimulus for TG2 activation. Following stimulation with the calcium ionophore, TG2 activity appeared throughout the cytosol, while cellular boundaries remained clearly visible (Figure 5). On the other hand, DTT induced TG2 activity at the cell membrane, producing a mirror image of the activation pattern with A23187. In addition to the TG2 activity at the membrane, patches of high TG2 activity were observed at the interface of the endothelium and smooth muscle layers (Video S1). Thus, a clear difference was observed in the localization of TG2 activity with A23187 and DTT.

### Intracellular reduction increases TG2 protein at the cell surface

**Figure 6:**
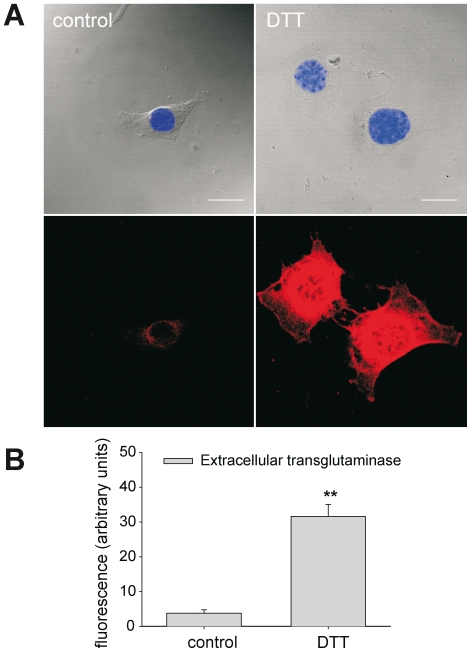
Intracellular reduction increases cell surface TG2. Incubation of cultured smooth muscle cells with DTT (0,1 mmol/L) increased the amount of extracellular TG2 (shown in red), as visualized by immunostaining of non-permeabilized cells. Fluorescence intensity was integrated per cell and averaged over 35–37 cells per group. Scalebar = 20 µm. ** non-stimulated vs. DTT: P<0.01.

The confocal images showed a membrane-bound TG2 activity upon stimulation with DTT, but did not provide sufficient resolution to determine whether the activity is intra- or extracellular. We therefore studied the presence of TG2 on non-permeabilized smooth muscle cells with immunocytochemistry. Cultured SMCs were stimulated with a low dose of DTT and stained for extracellular TG2. This revealed a strong increase in the level of extracellular TG2 as compared to untreated control cells (Figure 6). Hence, these data suggest that a reduced state triggers translocation of TG2 from the cytosol to the cell surface.

### Substrates of TG2

As TG2 activity at the smooth muscle cell membrane was associated with inward remodeling, we next investigated the extracellular substrates of TG2 using cultured SMCs that were stimulated with DTT. Labeling of TG2 substrates with the lysine donor biotinylated pentylamine (BPA) or a specifically designed Q-peptide (glutamine donor) was followed by a pull down assay and mass spectrometry. This revealed a number of proteins as substrate for TG2 (Table 1). Fibronectin was the major extracellular substrate, cross-linked both to BPA and Q-peptide. In addition, BPA identified the ECM components collagen, fibulin-2 and nidogen-1 as glutamine donors for transamidation. Both fibronectin and nidogen-1 were subsequently confirmed as TG2 substrates in western blots (Figure S3). Furthermore, a number of cytoskeletal and other intracellular proteins were identified.

**Table 1:** TG2 substrates in cultured smooth muscle cells.

	control	BPA	Q-peptide	known
Extracellular Matrix				substrate 1
fibronectin precursor		63	47	yes
collagen alpha-1 chain precursor		4	1	yes
fibulin-2 precursor		2		no
nidogen-1 precursor		2		yes
**Cytoskeleton & Cell Membrane**				
vimentin	6	14	13	yes
actin	3	14	12	yes
tubulin alpha-1	1	7	5	no
tubulin beta-5		7	3	yes
moesin		4	2	no
WD repeat protein 1		2		no
annexin A1			2	no
V-CAM1		3		no
**Cell Metabolism**				
alpha-enolase	1	10	2	yes
L-lactate dehydrogenase	2	10	6	no
pyruvate kinase		9	2	no
GAPDH	4	4	6	no
aldehyde dehydrogenase, mitochondrial		2	1	no
voltage-dependent anion-selective channel		2	1	no
transketolase		2		no
**DNA, RNA & Protein Synthesis**				
elongation factor 1		7	4	yes
histone H1.2		5		yes
THO complex	4	3	4	no
initiation factor 4A-I	1	3	3	no
ribosomal protein 53-A		2	3	no
elongation factor 2		1	3	no
**Various**				
heat shock protein 90		7	4	yes
guanine nucleotide-binding protein		5	3	no
serum albumin precursor		1	4	no
multifunctional protein ADE2			2	no

Cultured smooth muscle cells were incubated with BPA and Q-peptide, which were used as lysine and glutamine donor respectively. Samples were purified by a streptavidin pull down assay and bands were analyzed by mass spectrometry. Values represent the number of unique peptides found for each protein and give an indication of the abundance of the protein in the sample.

1 *Database used:* http://genomics.dote.hu/wiki/index.php/Category:Tissue_transglutaminase.

### Regulators of redox balance during in vivo remodeling

The activation of TG2 by DTT proved to be a strong stimulus for inward remodeling, but represents an artificial means of manipulating the redox state. Therefore, the expression of several enzymes capable of reducing thiol groups on proteins was studied in vessels remodeling in vivo. The mRNA expression was assessed in vessels stimulated to remodel inwardly by reducing blood flow in vivo. In arteries undergoing inward remodeling, selenoprotein P upregulation peaked after 2 days (Table 2). Glutathione transferase was already increased 80% after 24 hrs, followed by a gradual decline of expression. In outward remodeling vessels on the other hand, these enzymes were downregulated after 1 and 2 days (data not shown).

**Table 2:** Changes in mRNA of redox regulating enzymes during inward remodeling.

	Day			repetitions
	1	2	4	on arrays
Glutathione Reductase	40	30	10	*1*
Glutathione Synthetase	10	7	30	*3*
Glutathione Transferase T1	80	50	−20	*2*
Macrophage Migration Inhibitory Factor	57	13	70	*3*
Protein Disulphide Isomerase	0	−10	20	*1*
Selenoprotein P	28	174	62	*5*
Thioredoxin	−30	−70	0	*1*
Thioredoxin Reductase	20	20	20	*1*
Xanthine Dehydrogenase	73	90	47	*3*

Inward remodeling was induced by a surgically imposed decrease in blood flow in rat mesenteric arteries. Vessels were harvested at several time points and mRNA expression was determined by microarray analysis. Data represent changes in mRNA expression as percentage from control. An increased expression of several redox regulating enzymes was found. For each time point, vessels from 4 animals were pooled. Some genes were present several times on the microarray.

## Discussion

Vascular remodeling of small arteries after reduced blood flow, hypertension, and exposure to vasoconstrictors depends on TG2 [7], [8], [11]. In large arteries TG2 is involved in vascular calcification, and atherosclerotic plaque development and stability [27]–[31]. Recently, also large artery stiffening associated with aging was shown to be dependent on TG2 [15]. While many studies have contributed to the understanding of the regulation and functions of TG2 [13], the actual role of TG2 in vascular remodeling remained largely unknown. In our previous work we identified a strong relationship between vascular tone and remodeling. We reported that persistent vasoconstriction induces inward remodeling in several types of arteries [11], [32], [33]. This remodeling could be inhibited or reversed by vasodilator compounds such as the calcium channel inhibitors verapamil [32] or amlodipine [7]. As constriction and dilation mechanisms act partly through modulation of intracellular calcium levels, we herein tested the hypothesis that elevation of intracellular calcium triggers TG2 activity and remodeling. The calcium ionophore indeed increased intracellular transglutaminase cross-linking activity, but remodeling was completely absent. Thus, although intracellular proteins might have been cross-linked, this did not affect vessel caliber (Figure 4). Thus, these results suggested that other mechanisms of TG2 activation than elevation of intracellular calcium operate during vascular remodeling.

We found that the redox state of TG2 is a critical determinant in small artery remodeling. DTT induced a strong inward remodeling response, which was inhibited by the TG2 inhibitor (Figure 2). The data on remodeling by exogenous recombinant TG2 (Figure 1) underline the need for a reduced environment. As the cell-impermeable reducing agent TCEP did not change blood vessel diameter, the remodeling most likely stems from activation of an intracellular source of TG2. Since transglutaminase activity associated with remodeling was located at the surface of smooth muscle cells, we inferred that upon intracellular reduction, TG2 is excreted but remains bound to the cell membrane. This was substantiated by specific staining of extracellular TG2, using cultured SMCs. Similar mechanisms may exist in endothelial cells [15]. We speculate that after translocation to the cell membrane, TG2 activity is fully uncovered by the high extracellular calcium concentration.

The reversible formation of disulfide bridges, S-nitrosylation and S-glutathiolation of key cysteines are increasingly recognized as crucial mediators of protein activation and function [34]–[36]. TG2 is a good example of such regulation. It contains 18 sulfhydryl residues which can potentially be oxidized to form disulfide bridges, thereby impairing TG2 function [23], [37], [38]. The active site cysteine (Cys-277) was shown not to be easily prone to form disulfide bridges, probably due to the low accessibility of the active cysteine [23], [37], [39], [40]. However, disulfide bonds formed using cysteine residues from other parts of TG2 have been shown to strongly reduce cross-linking activity as well [37], [40], [41]. Recently, a crucial cysteine pair at amino acids 370–371, controlled by Cys-230, was identified [23]. It has been hypothesized that these disulfide bridges impede the calcium-triggered conformational change that is required for activation [23], [37]. Thus, a reduced state is necessary for TG2 to allow its cross-linking action. Generally speaking, the intracellular compartment is relatively reduced, whereas the extracellular environment is more oxidized [42]. More detailed studies have revealed that the redox balance is further controlled at the subcellular level [42]–[44]. In addition, the redox state of proteins can be individually regulated [45]. The cell-permeable reductant DTT that we used is known to activate in situ transamidation without affecting other TG2 functions [46]. We recognize that DTT is a non-specific reducing agent that might exert toxic effects. However, incubation with DTT did not affect vessel viability, assessed by its ability to contract (Figure S4). Hence, DTT did not have an overt detrimental effect on the used ex vivo vessels. In addition, the concentration of DTT that was used appears relatively high, but one needs to keep in mind that intracellular reducing compounds such as glutathione are present in the millimolar range [14].

The observation that a reduced state of TG2 is essential for its cross-linking activity, seems to contradict a report that TG2 is activated by oxidative stress via reactive oxygen species (ROS) [47]. However, UV irradiation or administration of exogenous H_2_O_2_ did not potentiate in vitro TG activity in a large number of cell types [14], [48]. Therefore, TG2 activity in response to ROS may be a secondary effect, possibly linked to calcium leakage over the damaged cell membrane or increased TG2 expression in apoptosis-prone cells [14], [49], [50]. Indeed, cytosolic ROS were reported to trigger an increase in cytosolic calcium [42]. In addition, generation of ROS was reported to inhibit TG2 degradation [51].

In addition to disulfide formation, TG2 cross-linking activity can be regulated by nitrosylation of cysteines [38]. Here, the redox balance plays an important role as well: cationic nitric oxide causes S-nitrosylation, but anionic NO leads to formation of a disulfide bridge [52]. In the present study, we showed that activation of TG2 within the vessel wall, either by reduction or elevated intracellular calcium is inhibited by NO. The inhibition of TG2 activity fully prevented inward remodeling. As the nitric oxide level falls in several physiological and pathological conditions, such as low blood flow, hypertension and aging, this may be an important determinant in transglutaminase activity in vivo. Indeed, we previously showed that inhibition of NO synthesis results in TG2 dependent inward remodeling [1], [53]. In further support, recent work by Santhanam et al. [15] showed that vascular stiffening associated with aging depends on TG2 activity and a reduction in nitric oxide levels.

The substrates of TG2 in small artery remodeling have not been previously studied. We identified fibronectin as both a lysine and glutamine donor (Table 1). Another relevant extracellular substrate is fibulin-2, which is active in tissue remodeling by cross-linking several elements in the pericellular matrix [54]. The confocal images showed high TG2 activity at the interface between smooth muscle cells and endothelial cells (Video S1). This layer, the internal elastic lamina, contains laminin-nidogen complexes and collagen type IV, which are both known substrates for TG2 [55], [56]. We confirmed nidogen-1 as substrate for SMC-derived TG2 using BPA. Regarding the collagen family, the alpha-1 chain of collagen type I was detected as TG2 substrate. Thus, although we identified a number of proteins that are cross-linked and known to determine extracellular matrix properties, the actual substrate(s) responsible for remodeling remains to be determined. Also, the relevance of the intracellular substrates, including cytoskeletal proteins and glycolytic enzymes, warrants further investigation.

An important question is how reduction of specific proteins such as TG2 is achieved in vivo. We addressed this question by studying mRNA expression of redox-related proteins using a micro-array approach. Here we found several reducing enzymes to be quickly upregulated in vessels undergoing inward remodeling (Table 2). Glutathione transferase (GST), which catalyzes the conjugation of reduced glutathione to substrate proteins, was strongly upregulated after 1 and 2 days, together with glutathione reductase. Interestingly, a broad protein interaction study identified GST as the 2^nd^ most important interaction partner of immobilized TG2 [56], a finding confirmed in several pathological conditions [57]–[59]. Thioredoxin is expressed amongst others in endothelial and SMCs, was detected in plasma [60], [61] and is involved in protein denitrosylation [34]. Surprisingly, thioredoxin was downregulated in inward remodeling vessels, although its corresponding reductase was slightly upregulated at all time points. On the other hand, the expression of selenoprotein P, which is structurally closely related to thioredoxin reductase, was elevated 174% after 2 days. Although protein disulfide isomerase was reported to act as reductase at the cell membrane of SMCs [62], its expression level was unchanged. Macrophage migration inhibitory factor, which plays a role during inflammation but also in redox regulation at the cell membrane [63], was upregulated at all time points. Taken together, the upregulation of a panel of reducing enzymes was identified in vessels in the process of inward remodeling. These enzymes could provide a more reduced state necessary for TG2 cross-linking activity.

In summary, using the Cys-277 mutant of TG2 and an active-site inhibitor, we showed that the cross-linking action of TG2 is necessary to induce inward remodeling of small arteries. Importantly, TG2 needs to be kept in a reduced state to fulfill its action. Within the vessel wall, we found that smooth muscle cells respond to intracellular reduction with a strong increase in TG2 activity at the cell membrane. This localized transglutaminase activity was associated with inward remodeling. Of physiological and pathological relevance, TG2 activity and remodeling could be inhibited by addition of a NO-donor. A schematic overview of the results is given in Figure S5. Using mass spectrometry, we identified a number of proteins as substrates for TG2 that could be responsible for the change in vessel caliber. Finally, we found several reducing enzymes that were strongly upregulated in vessels undergoing inward remodeling induced by low blood flow. These enzymes could help to provide a more reduced state during inward remodeling in vivo.

## Supporting Information

Figure S1 **Effect of DTT on arteries from TG2 knockout mice.** Passive pressure-diameter relationships of isolated, cannulated arteries from TG2 knockout mice that were exposed to DTT showed inward remodeling. In contrast to arteries from C57BL/6 mice, the remodeling of arteries from TG2 knockout mice was not sensitive to the TG2 inhibitor L682777. *** Indicates P<0.001 for control vs. DTT.
(TIF)

Figure S2 **SNAP does not alter vessel properties.** Passive pressure-diameter relationships of isolated, cannulated arteries were not altered after 24 h exposure to the nitric oxide donor SNAP (n = 5).
(TIF)

Figure S3 **Fibronectin and nidogen-1 are substrates for TG2 in DTT treated smooth muscle cells.** TG2 substrates were labeled with BPA or Q-peptide and subsequently purified by streptavidin pull down. Control cells were treated with DTT only. (A) Coomassie staining of the total lysates and pull downs (* indicates streptavidin band). (B) Western blot stained with antibodies against fibronectin and nidogen-1 shows specific labeling of both proteins with the lysine donor BPA. For fibronectin an additional high molecular weight product can be observed which is labeled with both the lysine and the glutamine donor substrate.
(TIF)

Figure S4 **Effect of DTT on vessel reactivity.** Treatment with DTT did not significantly alter the vessel response to the thromboxane analogue U46619. After incubating vessels for 24 hrs with 2 mmol/L DTT, maximal contraction to U46619 was unchanged. Data are expressed as percentage of vessel diameter at full relaxation.
(TIF)

Figure S5 **Schematic overview of experimental results.** Exogenous, recombinant TG2 required a reducing agent to induce inward remodeling, which was accomplished by cross-linking. Only a cell-permeable reducing agent activates a pool of endogenous TG2 that can induce inward remodeling. Intracellular TG2 translocates to the cell surface and subsequently cross-links a number of proteins. This can be prevented with a site-specific inhibitor of TG2 or an NO donor. Exposure to calcium ionophore increases intracellular transglutaminase activity, which also can be counteracted with SNAP. In this case, however, inward remodeling is absent.
(TIF)

Video S1 **TG2 activity in the vessel wall.** TG2 activity was visualized by incorporation of AlexaFluor594/cadaverine and scanned by confocal microscopy over different layers of the blood vessel. TG2 activity was low in the intima and adventitia. Smooth muscle cells in the medial layer, aligned perpendicular to the vessel long axis, display strong membrane-bound TG2 activity. In addition, patches of transglutaminase activity were observed between the endothelium and smooth muscle layer.
(AVI)
