# Supplementary figures and images for: The Redox State of Transglutaminase 2 Controls Arterial Remodeling
Source: PLoS One. 2011 Aug 25;6(8):e23067. doi: 10.1371/journal.pone.0023067 (PMC3161997; doi:10.1371/journal.pone.0023067)

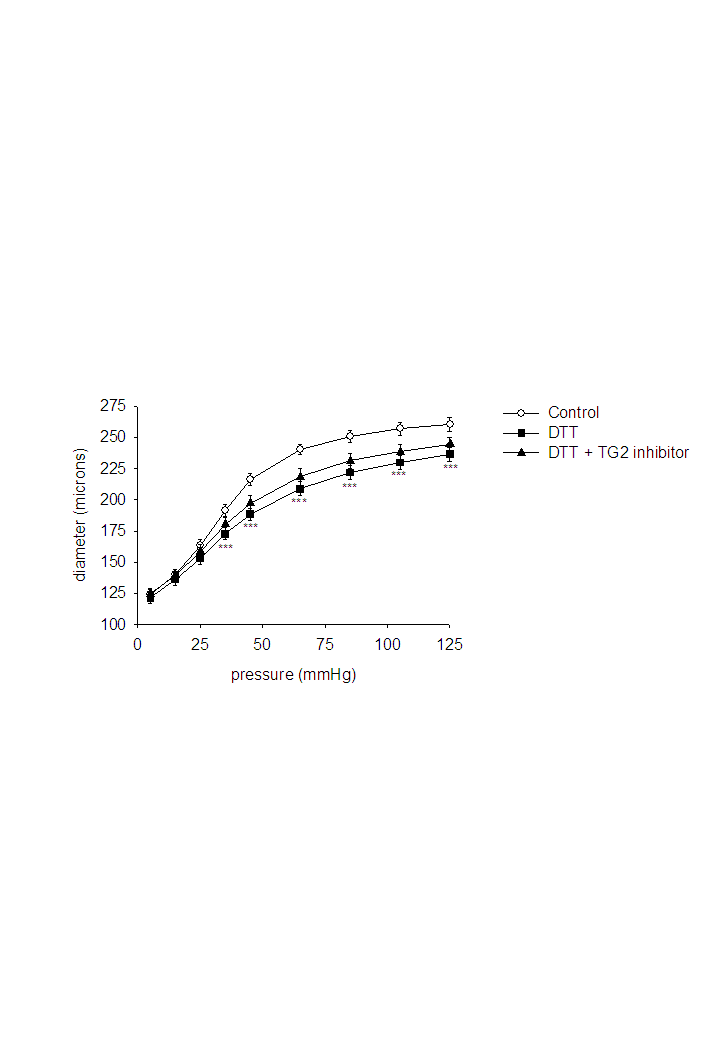

Supplement: Figure S1 — Effect of DTT on arteries from TG2 knockout mice. Passive pressure-diameter relationships of isolated, cannulated arteries from TG2 knockout mice that were exposed to DTT showed inward remodeling. In contrast to arteries from C57BL/6 mice, the remodeling of arteries from TG2 knockout mice was not sensitive to the TG2 inhibitor L682777. *** Indicates P<0.001 for control vs. DTT. (TIF) [file pone.0023067.s001.tif]

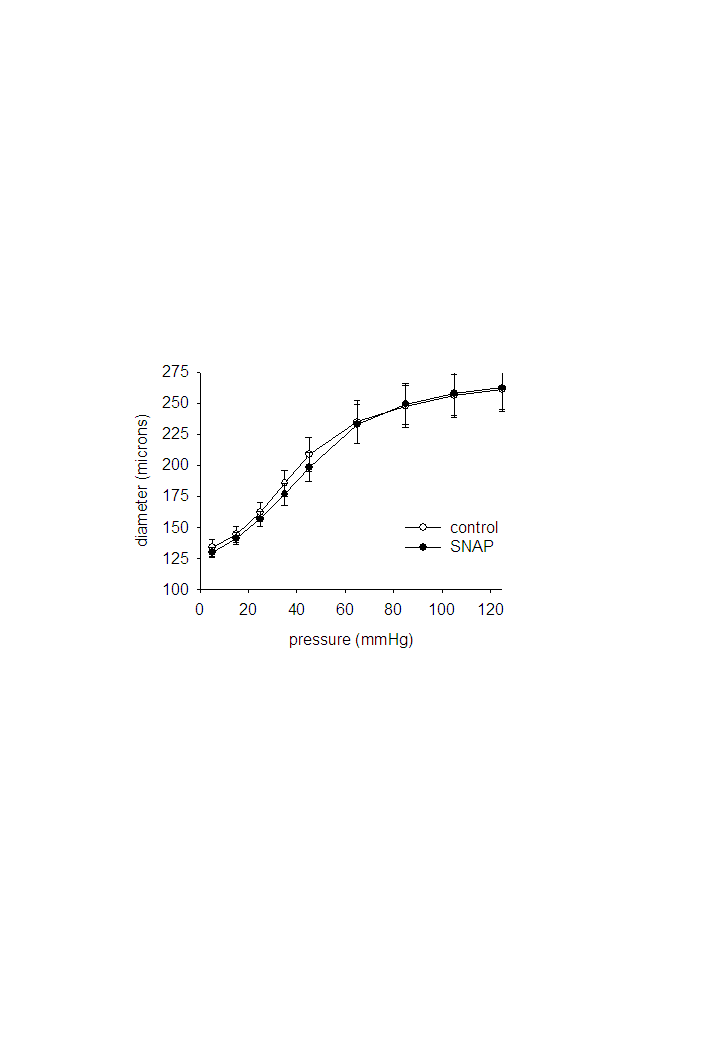

Supplement: Figure S2 — SNAP does not alter vessel properties. Passive pressure-diameter relationships of isolated, cannulated arteries were not altered after 24 h exposure to the nitric oxide donor SNAP (n = 5). (TIF) [file pone.0023067.s002.tif]
